# Prevalence trends of oral squamous cell carcinoma. Mexico City’s General Hospital experience

**DOI:** 10.4317/medoral.18043

**Published:** 2013-02-05

**Authors:** Juan C. Hernández-Guerrero, Luís F. Jacinto-Alemán, María D. Jiménez-Farfán, Alejandro Macario-Hernández, Florentino Hernández-Flores, Avissai Alcántara-Vázquez

**Affiliations:** 1 DDS, MSc, PhD, Immunology Laboratory, Postgraduate and Research Division, Dental School, National Autonomous University of Mexico, Mexico, D.F., MEXICO; 2 DDS, PhD, Immunology Laboratory, Postgraduate and Research Division, Dental School, National Autonomous University of Mexico, Mexico, D.F., MEXICO; 3 DDS, Immunology Laboratory, Postgraduate and Research Division, Dental School, National Autonomous University of Mexico, Mexico, D.F., MEXICO; 4 DDS, Maxilofacial Surgery Department, Dental School, National Autonomous University of Mexico, Mexico, D.F., MEXICO; 5 MD, Pathology Departament, Mexico City’s General Hospital, SSA, Mexico, D.F., MEXICO

## Abstract

Objective: Recent reports suggest an increase in oral squamous cell carcinoma (OSCC) frequency. To improve programs in public health, it is necessary to understand the epidemiological conditions. The aim of this study was to analyze the trend in gender, age, anatomic zone and OSCC stage from Mexico City’s General Hospital patients from 1990 to 2008. Study design: A retrospective review of all OSCC cases diagnosed by the Pathology Department of the Mexico City General Hospital was performed. Demographic data, in addition to anatomic zone and histological degree of differentiation were obtained. Central tendency, dispersion and prevalence rate per 100,000 individuals were determined. Results: A total of 531 patients were diagnosed with OSCC; 58.4% were men, giving a male:female ratio of 1.4:1, and the mean age was 62.5 ± 14.9 years. The predominant anatomic zone was the tongue (44.7%), followed by the lips (21.2%) and gums (20.5%). The most frequent histological degree was moderately differentiated in 325 cases (61.2%). The rates of OSCC prevalence showed similar patterns in terms across time. A significant correlation (P = 0.007) between anatomic zone and age was observed. Conclusion: According to our results, the prevalence of OSCC does not show important variations; however, a relationship between age and anatomic zone was observed. These data could be used as parameters for the diagnosis of OSCC as well as for the development and dissemination of preventive programs for the early detection of oral cancer.

** Key words:**Oral squamous cell carcinoma, prevalence, histology degree and anatomic zone.

## Introduction

Currently, cancer is one of the most common causes of morbidity and mortality. It has been estimated that there were approximately 12.7 million new cases worldwide and more than 7.6 million deaths in 2008 (1). It is projected that by 2020, every year there will be 15 million new cancer cases and 10 million cancer deaths.

Alcohol consumption, tobacco smoking, unhealthy diets, sedentary lifestyles, and viral infections are risk factors for cancer development. Drinking alcohol and smoking tobacco can synergistically cause cancer of the oral cavity, pharynx, larynx, and esophagus (2,3). While smoking prevalence is declining in economically developed countries, it is increasing in some developing countries in South America, Asia, and Africa (2).

Oral cancer is a public health problem, representing the sixth most common malignant neoplasm. The annual estimated incidence is approximately 275,000 oral cancers; two thirds of these cases occur in developing countries (4). In 2008, Globocan indicated that 1.8% of all neoplasms in Mexico (all cancers, excluding non-melanoma skin cancer, 127,604 tumors) are oral cancers (5). The oral squamous cell carcinoma (OSCC) represents more than 90% of malignant neoplasms of the mouth (6). Their principal anatomic zones are the tongue, floor of the mouth, gums, palate, oral mucosa, and other sites in the mouth. The anatomic zones or sites affected vary based on geographical areas. For example, the tongue is the most common site for intraoral cancer among Europeans and in the US population, accounting for 40-50% of oral cancers, whereas buccal cancer is more common among Asian populations due to betel quid/tobacco chewing habits. However, data on the trends for OSCC in Mexican and Latin-American populations are scarce (4). OSCC has a propensity for early and extensive lymph node metastases and predominantly occurs in alcohol drinkers and tobacco smokers in their 5th and 6th decades of life (6,7). Optimal therapy and survival from oral cancer depend on adequate diagnosis and assessment of the primary tumor and its clinical extent. The clinical diagnosis must be confirmed by biopsy and histological analysis. The relevant histological features include the loss of the basement membrane and disturbances in architectural and cytological epithelium features with invasion of the connective tissue (7,8). The aim of the present study was to analyze the trend in gender, age, anatomic zone and OSCC stage from Mexico City’s General Hospital patients from 1990 to 2008.

## Material and Methods

A retrospective review of all OSCC archives from the Pathology Diagnosis Department of the General Hospital of Mexico (GHM), Mexican Health Ministry, from 1990 to 2008 was performed after obtaining Institutional Research and Ethics Board approval. Demographic, clinical and histological data, such as age, gender, anatomic zone, and histological differentiation degree, were obtained. Anatomical zones were classified according to the International Statistical Classification of Diseases and related health problems, which covers cancer of the lips (C0.00-C00.9), tongue (C02.0-C02.3), gums (C03.0-C03.9), floor of the mouth (C04.0-C04.1), palate (C05.0), and other unspecified (C06.0-C06.2) parts of the mouth (9). The histological differentiation degree was classified as well differentiated (WD), moderately differentiated (MD), or poorly differentiated (PD) according to the World Health Organization WHO guidelines (7).

Trend analyses of OSCC patients who were less than 40 years old and those who were older than 40 years old were performed. In addition, data were analyzed in periods: 1990-1994, 1995-1999, 2000-2004, and 2005-2008. The means and standard deviations were calculated. The prevalence rate of OSCC per 100,000 individuals was calculated according to the metropolitan zone population obtained from the National Population Census (10). Spearman’s correlation test was used to determine the relationship between variables. The SPSS 13.0 software (IBM, Chicago, Il) was used for statistical analysis.

## Results

A total of 531 patients diagnosed with OSCC were registered from 1990 to 2008. The mean age was 62.5 ± 14.9 ranging from 16 to 98 years of age; the distribution of patients less than 40 years old represented 11.6% (62 patients) and those more than 40 years old represented 88.3% (469 patients). Overall, 58.4% (310 patients) of the patients were men, and 41.6% (221 patients) were women, giving a male:female ratio of 1.4:1.

The most frequent anatomical zone reported was the tongue at 44.7% (237 cases), followed by the lips at 21.2% (113 cases), the gums at 20.5% (109 cases), the floor of the mouth at 8.9% (47 cases), palate at 0.5% (3 cases) and others sites of mouth at 4.2 % (22 cases). Based on WHO guidelines, the histological differentiation degree of all OSCC cases was as follows: 61.2% (325 cases) corresponded to MD, 19% (101 cases) corresponded to WD, and 13.6% (72 cases) corresponded to PD. Only 6.2% (33 cases) were diagnosed as carcinoma in situ ( Table 1).

**Table 1:**
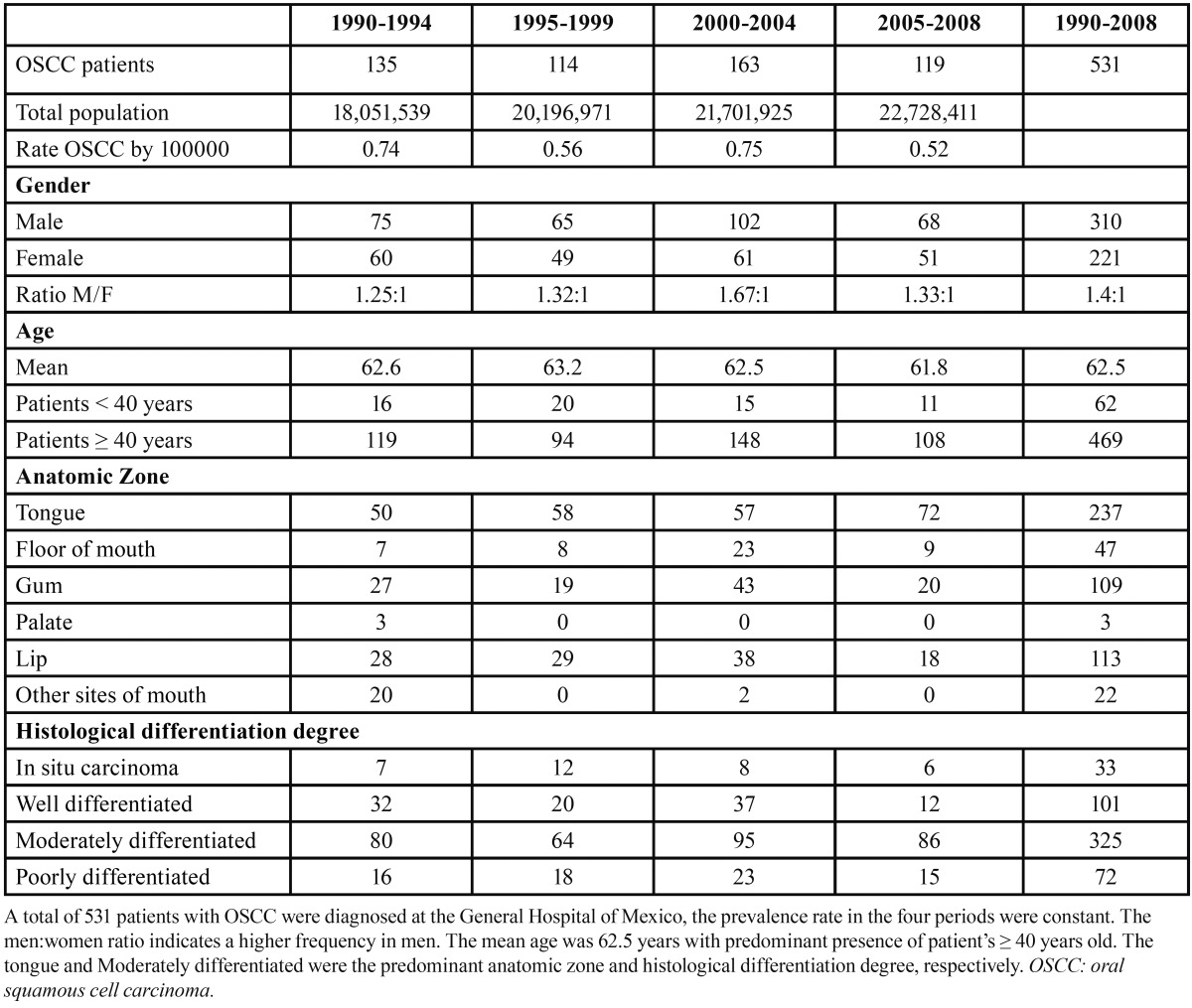
Periodic and general demographic, clinical, and histological data of OSCC patients.

The 1990 to 1994 period presented 135 patients with a rate of 0.74 OSCC cases per 100,000 inhabitants. The male:female ratio was 1.25:1. The mean age was 62.6 years, and 119 patients were ?40 years old. The predominant anatomic zone was the tongue, followed by the lips and gums, and the histological differentiation degree showed a predominant frequency of the MD degree ( Table 1). Comparing the above period to the 1995 to 1999 period, we observed a slight decrease in OSCC prevalence (0.56 OSCC cases per 100,000 habitants), whereas male:female ratio, age, gender, and histological differentiation degree showed similar patterns to the 1990 to 1994 period; we observed an increase in tongue frequencies and a decrease in gum frequencies. In the 2000 to 2004 period, we observed an increase in the OSCC prevalence rate (0.75 OSCC cases per 100,000 habitants) and the male:female ratio (1.67:1), and there were increase in the distribution of the anatomic zone (floor of mouth and gum). In the 2005 to 2008 period, we observed a decrease in the prevalence rate (0.52 OSCC cases per 100,000 habitants) and the male:female ratio (1.33:1). The ?40 years old patients were predominant in all periods. Based on anatomic zone, we observed that only the floor of mouth showed important differences in frequency with respect to gender. In relation to age and anatomic zone, a significant correlation (P = 0.007) was observed ( Table 2). The predominant histological differentiation degree was MD in all anatomic zones.

**Table 2:**
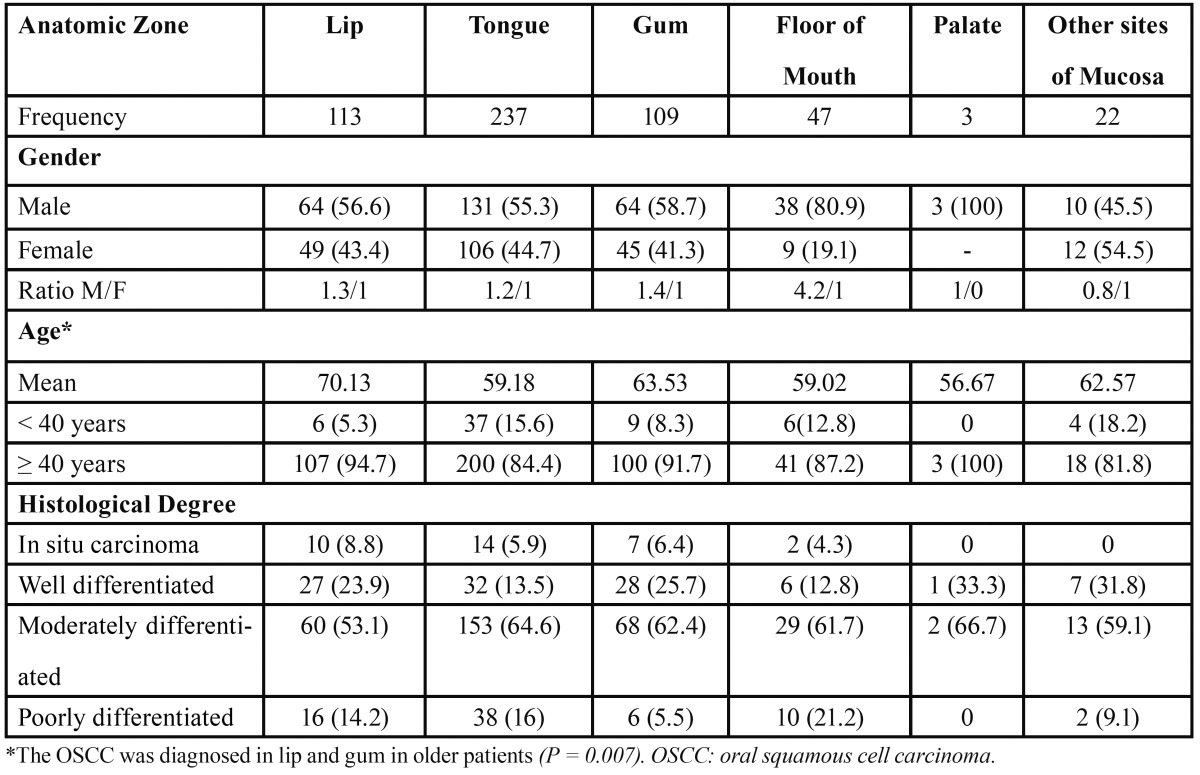
Anatomic zone of OSCC distribution.

## Discussion

In Mexico, there are few reports describing the national trends of OSCC incidence or prevalence (11-15). The complete understanding of the specifics of a disease requires an analysis of its epidemiological distribution, so that the necessary measures for its control or elimination can be implemented. Only from 2000 to 2008, the General Hospital of Mexico received approximately 654,270 patients per year; this number represents approximately 0.5% of the national population (10,16). Based on these numbers, the General Hospital could be considered as an epidemiological reference center because of its high patient volume. In the Oncology and Pathology departments of the hospital during 2000 to 2008, a total of 4925 patients were diagnosed with a neoplasm (16); during the same period, 282 OSCC cases were diagnosed, which represents approximately 5.7% of all neoplasms and suggests that oral cancer is an important health problem that requires the development and implementation of new programs for its control and prevention.

Our results indicate a mildly constant trend in OSCC prevalence. These results are controversial because they differ from the results reported by Gaitán-Cepeda et al. (11) who indicate that the OSCC has increased markedly; however, our results are consistent with those reported by Anaya-Saavedra et al. (12), who indicate that oral cancer mortality has not changed in two decades of study. Similar to our study, those studies were conducted in sufficiently large patient populations. Nevertheless, for an overall understanding of this disease in Mexico, a system for constant epidemiological monitoring of oral cancer and other types of neoplasm are required and would facilitate the control and prevention of cancer.

According to previous reports and our results, OSCC can be considered a public health problem. One possible reason for the prevalence of OSCC is tobacco and alcohol consumption. The National Addictions Survey 2008 showed a male:female ratio of alcohol consumption of 7.5:1, with a 5.5% alcohol dependence in the total population. Additionally, 35.6% of the population between 12 and 65 years of age (approximately 27 million people) has smoked at least once in their lives, and active smokers consume 7 cigarettes per day (17). The above data suggest that these behaviors are common and accepted by our population; however, in developed countries, both these cancer risk factors have shown a decrease, resulting in a decrease in oral cancer incidence (4). This highlights the need for tobacco and alcohol consumption prevention programs and an early diagnosis of oral cancer, which could impact the prevalence and incidence of this neoplasm.

In our population we observed a mean age of 62.5 ± 14.9 years; this observation is consistent with previous reports, which indicated that the risk of oral cancer development increases in relation to age, principally in patients older than 50 years (4,9,18). In recent years, reports indicate a substantial increase in numbers of young patients (less than 40 years old) with OSCC and suggest that this is related to Human Papiloma Virus (HPV) infections (19,20). Mannarini et al. has reported that the adult population has one or more HPV genotypes, suggesting that this virus could play an etiological role in the pathogenesis of potential malignant oral mucosa diseases and OSCC in young people (20). Our results indicate that only 11.7% of patients were diagnosed with OSCC before the age of 40 years, which could contradicts the previous theory that states HPV as important risk factor in young oral population; nevertheless, it would be interesting to determine the particular roll of this virus in oral carcinogenesis.

Historically, the association between gender and oral cancer has been reported more frequently in men than in women; this pattern had been attributed to men’s life style, alcohol and/or tobacco consumption and occupational risks; however, the male:female ratio has declined over the last decades (4). In Mexico, previous reports indicated a male:female ratio of 2:1, whereas our results indicate a 1.4:1 ratio. This difference could be attributed to the increased participation of women in risk factor behaviors, such as alcohol and tobacco consumption (14). If we consider that both genders have access or may be exposed to risk factors, the gender issue is secondary when risk behaviors are present.

The anatomic zone and histological differentiation degree are two important characteristics in OSCC; the biological behavior, treatment, and prognosis could be predicted based on both features (6-8). Our results from anatomic zone analyses indicated that the tongue, lips, and gums were the most affected zones. Sánchez-García et al. (15) and Durazzo et al. (21) indicated that the tongue was the principal anatomic zone affected in higher volume medical centers in Mexico and Brazil, respectively; however, reports from Asian populations indicate that buccal cancer was more frequent than that of other zones. The differences in anatomic zone frequency are attributed to differential behaviors in risk factors exposure, such as the use of cigarettes, cigars, and pipes or tobacco chewing (4,15,21).

A significant relationship between anatomic zone and age was observed; involvement of the lips, gums, and other sites of the mucosa were detected in patients who were older than 60 years, whereas OSCC involving the tongue, the floor of the mouth, and the palate was detected in patients younger than 60 years. The detection of OSCC may be associated with pain or functional alteration in the affect anatomic zone. The detection of age and anatomic zone could be associated with pain or functional alteration in the affected anatomical zone. In tongue OSCC, the tongue’s movement against the teeth causes discomfort, promoting attention by the patient. In contrast, lip and buccal mucosa carcinomas only show intense pain at advanced stages, allowing for a long period without medical or dental attention (6).

Definitive diagnosis of OSCC is obtained by histological analysis. The classification of tumor differentiation degree is performed to provide some indication of their aggressiveness, which may then be related to prognosis or treatment (7,8,22,23). Our results indicated that 325 OSCC were MD (moderately differentiated), followed by WD in 101 cases, and PD in 72 cases; only 33 cases were in situ carcinomas. Reports suggest that the aggressiveness of the neoplasm is inversely related to the differentiation degree; based on this concept, our results suggest that a substantial number of patients with MD carcinomas could present a limited or poor life quality and survival, however, additional parameters (TNM or stage) could be necessary to establish a better prognosis. Nevertheless, many reports suggest that the early diagnosis of oral cancer, based on pre-malignancies (leukoplakia or erythroplasia) and pre-cancer condition (dysplasia) detection is effective for adequate treatment and improved prognosis (24-26). Based on our results, only 33 patients were diagnosed with in situ carcinoma suggesting that a large portion of our sample seeks medical attention in the invasive stages of the disease.

Our data suggest that the OSCC trends were constant in the Mexican population from 1990 to 2008. The male:female ratio indicates that men present a higher frequency of the disease; however, if we consider the current attitudes in terms of alcohol and tobacco consumption in our population, we should consider these behaviors as the principal factors related to age, gender, and anatomic zone and, hence, potential risk of OSCC development. Whether the diagnosis is made early or is delayed determines whether oral cancer patients have a good or poor quality of life, respectively. The scarce epidemiology information on OSCC in our country makes it difficult to obtain a clear picture of the present situation. This study was based on information from a high volume public health care center in an attempt to increase our knowledge of OSCC and to develop more effective policies and strategies to address public health issues.

## References

[1] Ferlay J, Shin HR, Bray F, Forman D, Mathers C, Parkin DM (2010). Estimates of worldwide burden of cancer in 2008: GLOBOCAN 2008. Int J Cancer.

[2] Warnakulasuriya S, Sutherland G, Scully C (2005). Tobacco, oral cancer, and treatment of dependence. Oral Oncol.

[3] Petti S, Scully C (2005). Oral cancer: the association between nation-based alcohol-drinking profiles and oral cancer mortality. Oral Oncol.

[4] Warnalulasuriya S (2009). Global epidemiology of oral and oropharyngeal cancer. Oral Oncol.

[5] (2012). Cancer Incidence and Mortality Worldwide in 2008.

[6] Bagan J, Sarrion G, Jimenez Y (2010). Oral cancer: clinical features. Oral Oncol.

[7] Costa Ade L, Pereira JC, Nunes AA, Arruda Mde L (2002). Correlation between TNM classification, histological grading and anatomical location in oral squamous cell carcinoma. Pesqui Odontol Bras.

[8] Costa Ade L, AraÃjo JÃnior RF, Ramos CC (2005). Correlation between TNM classification and malignancy, histological features of oral squamous cell carcinoma. Braz J Otorhinolaryngol.

[9] MÃller S, Pan Y, Li R, Chi AC (2008). Changing trends in oral squamous cell carcinoma with particular reference to young patients: 1971-2006. The Emory University experience. Head Neck Pathol.

[10] (2011). Censo de Población y vivienda. Estados Unidos Mexicanos.

[11] GaitÃn-Cepeda LA, Peniche-Becerra AG, Quezada-Rivera D (2011). Trends in frequency and prevalence of oral cancer and oral squamous cell carcinoma in Mexicans. A 20 years retrospective study. Med Oral Patol Oral Cir Bucal.

[12] Anaya-Saavedra G, RamÃrez-Amador V, Irigoyen-Camacho ME, ZimbrÃn-Romero A, Zepeda-Zepeda MA (2008). Oral and pharyngeal cancer mortality rates in Mexico, 1979-2003. J Oral Pathol Med.

[13] Luna-Ortiz K, GÃemes-Meza A, Villavicencio-Valencia V, Mosqueda-Taylor A (2004). Lip cancer experience in Mexico. An 11-year retrospective study. Oral Oncol.

[14] Anaya-Saavedra G, RamÃrez-Amador V, Irigoyen-Camacho ME, GarcÃa-Cuellar CM, Guido-JimÃnez M, MÃndez-MartÃnez R (2008). High association of human papillomavirus infection with oral cancer: a case-control study. Arch Med Res.

[15] SÃnchez-GarcÃa S, JuÃrez-Cedillo T, Espinel-BermÃdez MC, Mould-Quevedo J, GÃmez-DantÃs H, de la Fuente-HernÃndez J (2008). Hospital discharges for oral cancer in the Mexican Institute of Social Security, 1991-2000. Rev Med Inst Mex Seguro Soc.

[16] (2011). Direccion de planeación y desarrollo de sistemas; Anuarios estadísticos..

[17] (2011). Cancer Incidence and Mortality Worldwide in 2008.

[18] Shiboski CH, Schmidt BL, Jordan RC (2005). Tongue and tonsil carcinoma: increasing trends in the U.S. population ages 20-44 years. Cancer.

[19] Al-Rawi NH, Talabani NG (2008). Squamous cell carcinoma of the oral cavity: a case series analysis of clinical presentation and histological grading of 1,425 cases from Iraq. Clin Oral Investig.

[20] Mannarini L, Kratochvil V, Calabrese L, Gomes Silva L, Morbini P, Betka J (2009). Human Papilloma Virus (HPV) in head and neck region: review of literature. Acta Otorhinolaryngol Ital.

[21] Durazzo MD, de Araujo CE, BrandÃo Neto J de S, Potenza Ade S, Costa P, Takeda F (2005). Clinical and epidemiological features of oral cancer in a medical school teaching hospital from 1994 to 2002: increasing incidence in women, predominance of advanced local disease, and low incidence of neck metastases. Clinics (Sao Paulo).

[22] Brandizzi D, Gandolfo M, Velazco ML, Cabrini RL, Lanfranchi HE (2008). Clinical features and evolution of oral cancer: A study of 274 cases in Buenos Aires, Argentina. Med Oral Patol Oral Cir Bucal.

[23] Losi-Guembarovski R, Menezes RP, Poliseli F, Chaves VN, Kuasne H, Leichsenring A (2009). Oral carcinoma epidemiology in ParanÃ State, Southern Brazil. Cad Saude Publica.

[24] Silverman S Jr, Gorsky M, Lozada F (1984). Oral leukoplakia and malignant transformation. A follow-up study of 257 patients. Cancer.

[25] Speight PM (2007). Update on oral epithelial dysplasia and progression to cancer. Head Neck Pathol.

[26] Zygogianni AG, Kyrgias G, Karakitsos P, Psyrri A, Kouvaris J, Kelekis N (2011). Oral squamous cell cancer: early detection and the role of alcohol and smoking. Head Neck Oncol.

